# Estimating the influence of field inventory sampling intensity on forest landscape model performance for determining high-severity wildfire risk

**DOI:** 10.1038/s41598-024-53359-8

**Published:** 2024-02-06

**Authors:** Hagar Hecht, Dan J. Krofcheck, Dennis Carril, Matthew D. Hurteau

**Affiliations:** 1 Spatial Informatics Group Natural Assets Lab, Pleasanton, CA USA; 2 Sandia National Laboratory, Albuquerque, NM USA; 3 US Forest Service, Santa Fe National Forest, Santa Fe, NM USA; 4 Department of Biology, University of New Mexico, Albuquerque, NM USA

**Keywords:** Ecology, Fire ecology, Forestry, Natural hazards, Fire ecology, Forest ecology, Forestry

## Abstract

Historically, fire has been essential in Southwestern US forests. However, a century of fire-exclusion and changing climate created forests which are more susceptible to uncharacteristically severe wildfires. Forest managers use a combination of thinning and prescribed burning to reduce forest density to help mitigate the risk of high-severity fires. These treatments are laborious and expensive, therefore optimizing their impact is crucial. Landscape simulation models can be useful in identifying high risk areas and assessing treatment effects, but uncertainties in these models can limit their utility in decision making. In this study we examined underlying uncertainties in the initial vegetation layer by leveraging a previous study from the Santa Fe fireshed and using new inventory plots from 111 stands to interpolate the initial forest conditions. We found that more inventory plots resulted in a different geographic distribution and wider range of the modelled biomass. This changed the location of areas with high probability of high-severity fires, shifting the optimal location for management. The increased range of biomass variability from using a larger number of plots to interpolate the initial vegetation layer also influenced ecosystem carbon dynamics, resulting in simulated forest conditions that had higher rates of carbon uptake. We conclude that the initial forest layer significantly affects fire and carbon dynamics and is dependent on both number of plots, and sufficient representation of the range of forest types and biomass density.

## Introduction

Fire has been an integral and essential part of Southwestern US forests for millennia^1,2^. Historically, the dry forests of the Southwest experienced frequent fire, which maintained their characteristic heterogeneous vegetation structure. However, a history of land-use and a policy of fire-exclusion over the past century has increased tree density, canopy continuity, and fuels, which has altered the way that fire behaves and interacts with the vegetation^3–5^. Additionally, a warming and drying atmosphere has been a catalyst for tree mortality, worsening the high fuel loads from fire-exclusion^6,7^ and making fuels more readily available to burn^8,9^. The combined effects of fire-exclusion and ongoing climate change have increased area burned and caused an increase in uncharacteristic fires, with large high-severity patches^10,11^. Large high-severity fires pose a challenge to society and ecosystems and have motivated societal investment in management activities that reduce the risk of high-severity wildfire^12^. However, the size of the area requiring restoration is large and efficiently deploying management resources requires understanding where on the landscape risk is greatest^13,14^.

Contemporary forest fuels management in the southwestern US involves a combination of thinning small diameter trees and prescribed burning to reduce tree density, surface fuels, and ladder fuels that can carry fire from the surface to the crown. This can reduce the risk of high-severity fire^15–17^. Yet, these treatments can have varying degrees of effectiveness for reducing the risk of high-severity fire and the per unit area costs range from tens to hundreds of dollars per hectare for prescribed burning to several thousand dollars per hectare for thinning and hand-piling the cut material (the most expensive treatment)^18–20^. One effective approach for reducing the risk of high-severity fire, especially in areas where consequences are greater (e.g. the wildland-urban interface), is thinning-from-below combined with prescribed burning. In the southwestern US, thinning combined with prescribed burning is one of the most expensive treatment combinations because many of the trees are not merchantable. Thus, preparing a landscape to receive fire and reduce high-severity wildfire risk is contingent upon using thinning treatments in a manner that they facilitate prescribed burning and managing natural ignitions for resource benefit over a larger fraction of the landscape^16,21^. Facilitating a return to a more heterogenous forest structure, maintained by frequent fire will help ensure a self-regulating system that keeps the available fuels limited^22^.

The goals of management vary in terms of desirable outcome and planned timeline, and are often a balancing act of multiple objectives that can include, in addition to fire severity reduction and forest restoration, watershed protection, habitat conservation, and carbon stabilization^23–26^. Achieving near-term objectives, such as reducing the risk of high-severity wildfire to communities, and long-term objectives, such as managing carbon storage to help regulate the climate, requires evaluation of trade-offs in both time and space. Accounting for trade-offs between management objectives across different temporal and spatial scales becomes increasingly difficult as the number of objectives and interactions between them increases, requiring the use of forest landscape models to better understand treatment scenarios and their possible outcomes^27–30^.

As resources available for management are limited, using them in the most advantageous way is crucial, and optimizing treatment placement has been another subject of forest landscape simulation studies^13,31,32^. In the case of wildfire, identifying landscape positions that have the highest probabilities of burning at high severity can help managers efficiently locate forest treatments. However, uncertainties in model output can limit their utility for decision-making. These uncertainties can be due to model structure^33^, key processes being absent from models^34^, or due to errors in the underlying data such as climate projections or the use of a generalized vegetation parameterization^35^. With all landscape vegetation models, the simulation is heavily influenced by the representation of vegetation conditions across the landscape. Uncertainty in the initial characterization of vegetation structure will likely propagate and compound with other sources of uncertainty as dependent processes are simulated in the model, such as vegetation competition, drought, and wildfire. These compounding uncertainties increase the challenges associated with using landscape models to inform decision making at any spatial or temporal scale, and therefore it is critical that we (1) develop a more rigorous understanding of how the uncertainty in the model inputs affects variability in model output, and (2) identify mechanisms to constrain that uncertainty with additional data.

In this study we evaluated the effects of forest inventory sample size on variability in the initial vegetation layer for an ecosystem model for the Santa Fe Fireshed in northern New Mexico. We leveraged prior research by Krofcheck et al.^29^ that evaluated thinning treatment placement optimization and its effects on forest carbon dynamics in the Santa Fe Fireshed and additional inventory data from the US Forest Service to quantify how the number of inventory plots influences model estimates of the high-severity wildfire. We compared our results to those of Krofcheck et al.^29^ to determine the effect of additional inventory data on forest treatment placement.

## Methods

### Study area

We conducted simulations in the Santa Fe fireshed, located in the Sangre De Cristo Mountains, east of Santa Fe, New Mexico. The fireshed is approximately 48,000 hectares (ha) and has an elevation ranging from 1900 to 3700 m. The prominent vegetation changes with elevation and is comprised of piñon-juniper (*Pinus edulis, Juniperus monosperma*) woodlands at lower elevations, ponderosa pine (*P. ponderosa*) at mid-elevations and mixed-conifer forest and spruce-fir (*Picea* spp., *Abies* spp.) at high-elevations. There are occurrences of gambel oak (*Quercus gambelii*) and quaking aspen (*Populus tremuloides*) stands in recently disturbed areas in the mid- and high-elevations. At lower elevations, the Sobordoro soils have a silty clay skeletal mixture, which transitions to loam-dominated soils at higher elevations. Mean climatic conditions over the period 1980–2015 included mean annual temperature of 9.4 °C and mean annual precipitation of 360 mm, with greater than 50% falling as snow in the winter months at higher elevations^36^.

### Initial communities data

**Figure 1:**
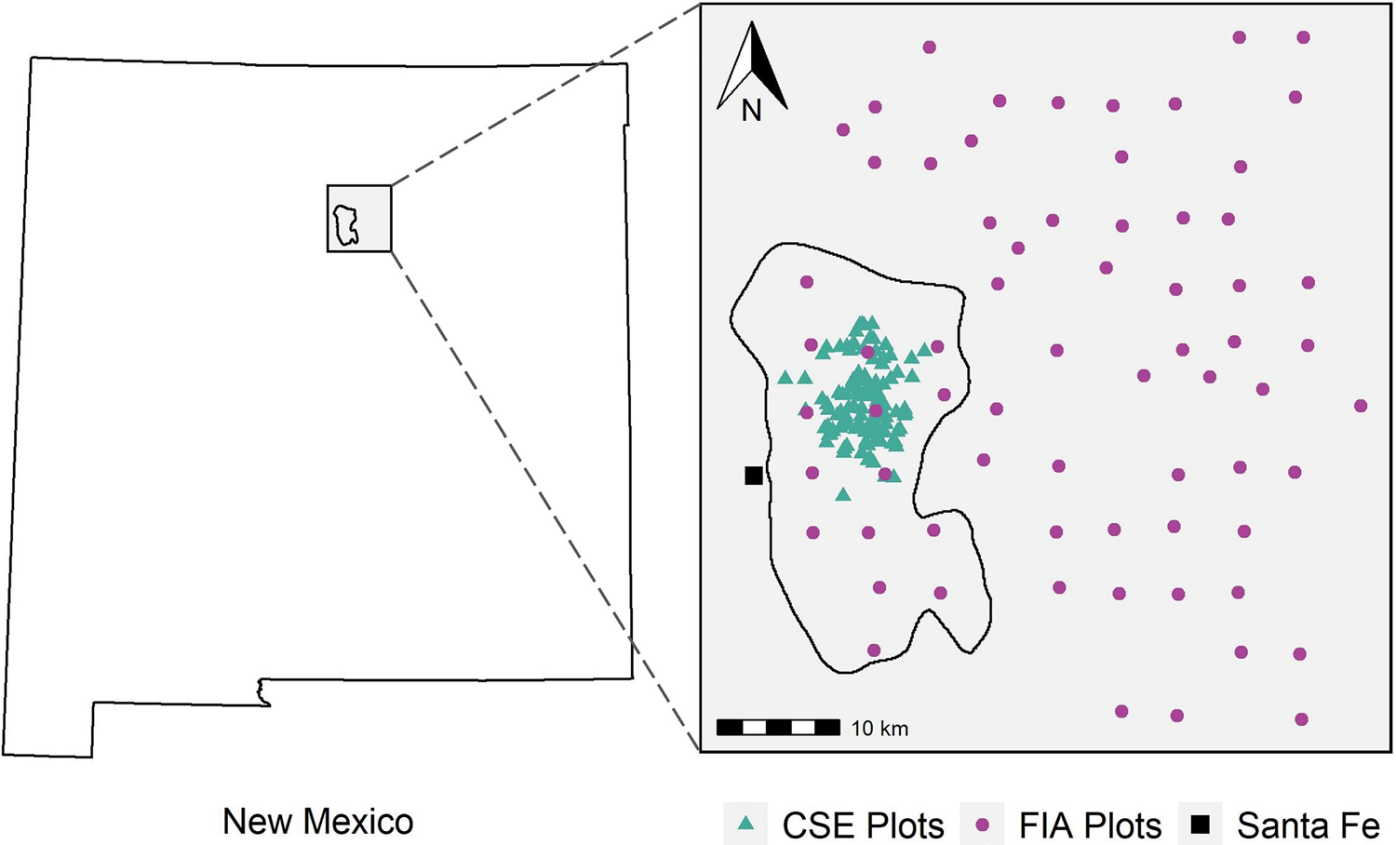
Study area location and boundary. Purple circles are the approximate Forest Inventory and Analysis (FIA) plot locations (n = 68) and the blue triangles are the Common Stand Exam (CSE) plots (n = 1072) represented by exact stand locations (n = 111). Each stand contains between 3 and 31 plots.

As with all forest landscape models, an initial characterization of forest conditions is required to initiate simulations. Here we used the LANDIS-II forest landscape disturbance and succession model. The initial communities layer is the base vegetation layer that sets the starting conditions for the exchange of carbon, water, energy, species interactions, disturbance effects, and other landscape processes. Given the importance of vegetation conditions for determining an optimal solution for thinning treatments, representing the spatial distribution of actual forest conditions well is central to generating simulation outputs that are useful to management decision-making. The initial treatment optimization study in this landscape^29^ used 68 Forest Inventory and Analysis (FIA) plots from within the Santa Fe National Forest that had been inventoried in 2010 or later and had not burned since 2005 (Fig. 1). Forest types represented by the FIA plots were piñon-juniper, ponderosa pine, Douglas-fir (*Pseudotsuga menziesii*), Engelman spruce (*Picea engelmannii*) and limber pine (*Pinus flexilis*). The latter three were grouped into a general mixed-conifer forest type. The authors then used elevation, transformed aspect using Topographic Radiation Aspect Index, TRASP^37^, and a tasseled cap transformation of spectral data from Landsat 8 (available at https://www.usgs.gov/landsat-missions/landsat-8) as predictors for Random Forest models and used the rfUtilities library^38^ to select the most parsimonious model for each forest category separately. Existing vegetation classification from the Southwest Regional Gap Analysis (SWReGap, https://swregap.org/) using the ‘yaImpute’ library^39^ was used to stratify the measured plots for the imputation. We determined plot sampling intensity by calculating the relative area of land each plot represents within its forest type.

To evaluate the influence of additional plot data on the initial communities layer and its effects on model behavior, we used data collected as part of the planning process by the US Forest Service. These data were located entirely within the study area and included 1072 plots from 111 stands inventoried in 2011, where each stand included between 3 and 31 plots (Fig. 1). The stands in this data set were sampled to support the Santa Fe Mountains Landscape Resilience Project and were primarily in ponderosa pine and mixed-conifer forest types (https://www.fs.usda.gov/project/?project=55088). Plot data were collected using a common stand exam protocol using variable radius plots. The specific Basal Area Factor (BAF) prism chosen for each stand was a function of stand density and they ranged from 10 to 30 BAF. We had coordinates for the centroid of each stand, but not for each individual plot, which affects the imputation process. For each imputation, we randomly selected one plot to represent the stand. We used the tree data from each plot to determine a specific forest type and generalized category (e.g. piñon-juniper, ponderosa pine, mixed-conifer), corresponding to the FIA classification, and added an aspen forest type, resulting in a total of four generalized forest types. We also defined non-forested areas and included two generalized species parameterizations to represent shrubs that resprout and shrubs that do not resprout following fire. We used all FIA and common stand exam (CSE) plot data (n = 1140) to generate a new initial communities layer following the same method as Krofcheck et al.^29^ in R v4.1.2^40^.

### Model and parametrizations

As the starting point, we used the species-specific parameters, fire and fuels parameters, and the ecoregion layer and parameters used in Krofcheck et al.^29^, and fixed all random seeds used to govern stochastic draws from distributions in the succession and wildfire components of the model. Our ultimate goal was to ensure that any differences we observed relative to the previously published work could be attributed to our experimental manipulations. The full modeling structure and parameterization are described below.

We used the Landscape Disturbance and Succession II (LANDIS-II) model^41^ with the photosynthesis and evapotranspiration (PnET) succession, Dynamic Fuels and Fire, and Biomass Harvest extensions to simulation forest growth and disturbance using a 100 m resolution. The core model simulates forest growth and succession for each grid cell using a demography-based approach to track species-specific age cohorts of biomass. Each species is parameterized independently with a unique set of parameters that govern their growth, succession, dispersal, and mortality across a spatially explicit landscape^41^. We used the (PnET) succession extension^42^, based on elements of the PNET-II model^43^. The PnET succession extension models carbon and water flux using species-specific physiological parameters. We used the parameters previously validated by Remy et al.^35^ for this area, which were obtained from previously published data and the TRY database, and validated against eddy covariance tower data^35,44,45^. We used the Dynamic Fuels and Fire extension^46^ to simulate wildfires. This extension was parameterized for the study area by Krofcheck et al.^29^ using regional fire size data from Geospatial Multi-Agency Coordination (https://rmgsc.cr.usgs.gov/outgoing/GeoMAC/historic_fire_data/), previously published fuels data^18,47–49^, and climate projections from the Multivariate Adaptive Constructed Analogs v2 collection to develop fire weather distributions (https://climate.northwestknowledge.net/MACA/). We used the Biomass and Harvest extension^50^ to simulate thinning treatments.

We used a cross between three elevation zones (https://datagateway.nrcs.usda.gov/) roughly corresponding to the vegetation transitions determined by the Southwest Regional Gap Analysis (https://swregap.org/) and six soil types (State Soil Geographic dataset, https://datagateway.nrcs.usda.gov/) to define 18 unique edaphic and climatic zones. These were used as ecoregions in the LANDIS-II core model and PnET succession extension. We used the same three elevation zones to define three different fire regions, which the model requires to define areas of similar fire weather, fire size distribution, and number of attempted ignitions.

We used monthly climate data, radiation, and atmospheric carbon dioxide concentration produced by Krofcheck et al.^29^. These were based on projections from the Localized Constructed Analogs statistically downscaled climate projection from five climate models forced with Representative Concentration Pathway 8.5 from the Coupled Model Inter-comparison Project Phase 5. The climate models chosen were Community Climate System Model (CCSM), Centre National de Recherches Météorologiques (CNRM), Flexible Global Ocean–Atmosphere-Land System Model (FGOALS), Geophysical Fluid Dynamics Laboratory (GFDL), and Model for Interdisciplinary Research on Climate (MIROC5-ESM 2) as their projections capture the range of temperature and precipitation for the study area.

### Simulation analysis

Given the importance of the initial communities layer for determining where high-severity fire probability is greatest on the landscape, we sought to estimate the uncertainty in the initial communities layer from not having coordinates for every CSE plot because the USFS data applies coordinates for a stand to all plots sampled in the stand. We also sought to estimate the uncertainty due to the number of plots used to interpolate the initial communities layer.

To estimate the uncertainty in the initial communities layer that is due to not having coordinates for all CSE plots, we re-ran interpolations randomly selecting one plot for each set of stand coordinates. We generated 31 initial communities layers, as this is the maximum number of plots in a single stand. We initiated simulations with each of these 31 initial communities layers using the five climate projections, for a total of 155 simulations. We compared the aboveground carbon following model initialization of these initial communities layers with the initial communities layer that we created using all plot data and that we used for our management simulations, henceforth referred to as the new layer. We calculated the difference in aboveground carbon between each layer and the one we used in our simulations to determine how much the initial communities layer is influenced by this source of uncertainty.

Inventory data can be costly to collect and limited data availability for developing the initial communities layer is a source of uncertainty that can influence identifying locations with a high probability of high-severity fire. To determine the influence of the number of plots used in the development of the initial communities layer, we produced five additional initial communities layers with differing numbers of underlying plot data. For four of the five layers, we halved the number of CSE plots used in the interpolation each time (e.g. 536, 268, 134, 67) and combined those with the FIA data. For the fifth initial communities layer, we only used the 68 FIA plots. For each of the layers, we randomly selected plots from each forest type proportional to its prevalence on the landscape. We then initialized the model using each of these initial communities layers using the five climate projections and compared the aboveground carbon following model initialization to that of the initial communities layer that we used for our simulations.

To compare our results with those of Krofcheck et al.^29^, we quantified differences between our primary initial communities layer and that of Krofcheck et al.^29^ by comparing the difference in quantity and distribution of aboveground carbon at the beginning and end of the simulations. We ran an independent t-test to assess the difference in carbon between the two studies in each grid cell every 10 years for each of the climate models, and computed the percent of area with a significant difference (p < 0.01) in aboveground carbon. We compared treatment location as determined by the probability of high-severity fire between our initial communities layer and that of Krofcheck et al.^29^. We calculated Net Ecosystem Carbon Balance (NECB) by subtracting carbon lost from the system (treatment and wildfires) from carbon gained (photosynthesis) and then relativized the treatment scenario NECB values to the no-management scenario for both our simulations and those of Krofcheck et al.^29^. Data processing and analysis was conducted using R v4.1.2^40^.

### Treatment scenarios

To develop the optimized treatment placement scenario, we first ran simulations that included no management to identify locations where landscape conditions were such that there was a high probability of high-severity wildfire. We ran the no-management simulations using the same five projected climate data sets and fire weather data described above. We ran 25 replicate simulations using each of the five projected climate data sets, for a total of 6250 simulation years. We used fire severity raster data from these model outputs to quantify the probability of high-severity conditional on a fire occurring by dividing the number of years with high-severity fires by the total number of years a fire occurred for each grid cell. The Dynamic Fire and Fuels Extension classifies fire severity using five classes, with classes four and five indicating that the majority of the overstory is killed. We treated fire severity values of four and five as high-severity. We then identified grid cells with a probability of high-severity fire greater than 0.3 and targeted those locations in the treatment scenario simulations, assigning treatment to those areas first.

To determine the type of treatment we used the probability of high-severity fire in combination with slope and forest type. Thinning and burning treatments were limited to the ponderosa pine and dry mixed-conifer forest where the combined ponderosa pine and Douglas-fir aboveground carbon was at least 65% of the total and treatments were only simulated on grid cells that had a probability of high-severity fire greater than 0.3. Thinning treatments were only applied to locations that had slopes < 30% to account for a common limitation on mechanical thinning. We simulated prescribed burning based on historic mean fire return intervals, with all ponderosa pine burned using a 10-year return interval and forests co-dominated by ponderosa pine and Douglas-fir burned using a 15-year return interval. We used the same thinning and prescribed burning treatments as Krofcheck et al.^29^, which were designed to approximate common treatments for the region. Thinning treatments simulated thinning from below by removing approximately 30% of the biomass, preferentially removing the youngest cohorts^48,51^.

To examine the effects of the treatment on the landscape we produced a new probability of high-severity fire raster and calculated the difference in aboveground carbon between the management and no management scenarios of this study at the end the simulations.

To generate the simulation output for comparison with the results from Krofcheck et al.^29^, we used the initial communities layer that was interpolated using the FIA data and CSE plot data from all 111 stands. We ran simulations over a 50-year period, using climate model projections for years 2000–2050. We ran 25 replicates for each of the five climate projections, totaling 125 simulations each for the no-management and management scenarios. Fire weather distributions tracked projected climate and were updated each decade to account for changes in temperature and precipitation.

## Results

### Initial communities

Our comparison of the 31 initial communities layers derived from randomly selecting plots from the forest stands sampled by the USFS indicated that the specific plots selected to represent each stand had a relatively small influence on aboveground carbon. The total aboveground carbon of the whole fireshed in teragrams (Tg) at year 1 of the simulations was similar when comparing simulations within each of the five climate models, and the median for all 155 simulations and across the five climate models was 3.601 Tg of carbon with an interquartile range (IQR) of 0.146 Tg. (Fig. S1a). We calculated the difference between the year 1 aboveground carbon of the initial communities layer we used for our management simulations (hereafter new layer) and the mean of year 1 aboveground carbon generated from replicate simulations of the 31 initial communities layers and found that only 560 hectares of the total 48,957 ha within the study area had a difference of more than 20 Megagrams per hectare (Mg ha^−1^) of carbon, the median of the difference was − 0.068 and the IQR was 0.859 Mg ha^−1^ of carbon (Fig. S1b). Typically, grid cells that had higher carbon values than those in the new layer were the aspen forest type and grid cells that had lower carbon values than the new layer were dominated by limber pine or ponderosa pine forest with a large limber pine component.

**Figure 2:**
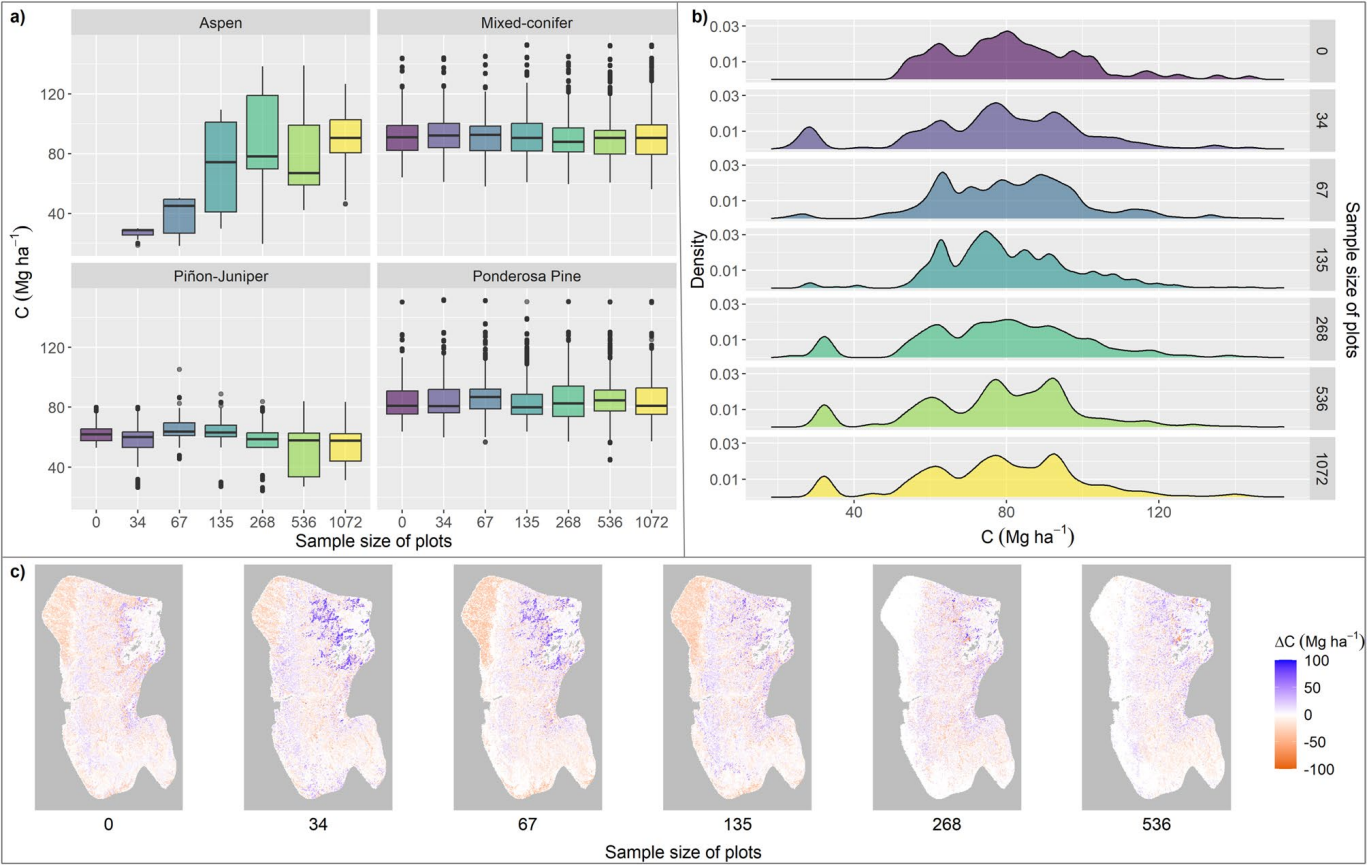
Biomass distribution of the different initial communities layers. (**a**) Boxplot of aboveground carbon per grid cell at year 1 of the simulation using different initial communities layers separated by forest type. X axis indicates number of available new plots in addition to 68 FIA plots used to create the initial communities layer. (**b**) Density distribution of aboveground carbon at year 1 of the simulations for each of the initial communities layers. (**c**) Difference in aboveground carbon at year 1 between the new initial communities layer developed using all 1072 plots and initial communities layers developed using fewer plots. Positive values indicate higher aboveground carbon in the initial communities layer developed using all 1072 plots. All values are in Megagrams of carbon per hectare.

The aboveground carbon distributions for ponderosa pine and mixed-conifer forests were fairly similar, regardless of the number of plots used to develop the initial communities (Fig. 2a). Estimates of aspen carbon differed substantially from the new layer, with median values decreasing by 12.22 Mg ha^−1^ or more (Fig. 2a). For piñon-juniper, median values were fairly consistent regardless of the number of plots used to develop the initial communities layer, but variability decreased substantially when fewer than half of the available plots were used (Fig. 2a), demonstrating the importance of adequate sampling to capture the variability in vegetation conditions (Fig. S2). The density distribution of aboveground carbon was similar for the new layer developed using all 1072 plots and the layer developed using half the plots. However, the layers that used fewer than half of the plots had decreased variability and were underestimating lower carbon grid cells of approximately 30 Mg C ha^−1^ and lower (Fig. 2b). The reduction in carbon variability with decreased numbers of plots used to inform the initial communities layer, when mapped spatially, shows that the largest discrepancies between the new layer and the others occurs in the vegetation types that are sampled less intensively than the more common ponderosa pine and mixed-conifer forest types (e.g. aspen and piñon-juniper) (Fig. 2c).

### Comparison of high-severity fire risk

**Figure 3:**
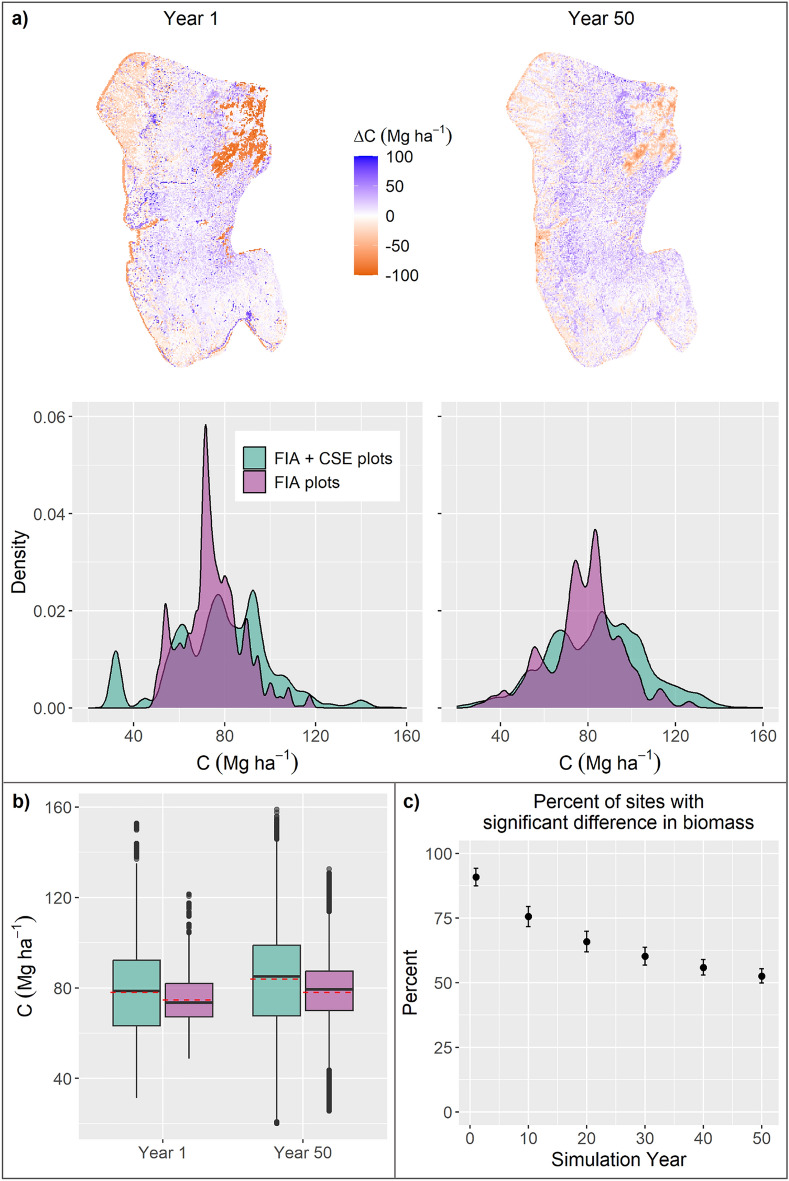
Difference in above ground carbon between the old (purple) and new (blue) initial communities layers. (**a**) Comparison of aboveground carbon when using FIA plots only (Krofcheck et al.^29^) and FIA + CSE plots (this study) for the no management scenarios. Top is the difference in aboveground carbon. Positive values indicate this study has a higher value than the previous study. Bottom is density plots. Left column is year 1 of the simulations and right column is year 50. (**b**) Boxplot of aboveground carbon in each of the studies for year 1 and 50. Red line is the mean. (**c**) Percent of grid cells with significantly different aboveground carbon (p < 0.01 for t-test). Points are mean for all climate models and error bars are standard deviation.

When we compared the model results from our initial communities layer developed using the 68 FIA plots in addition to the 1072 CSE plots (new layer) with those from the Krofcheck et al.^29^ initial communities layer developed using only the FIA plots (hereafter ‘old layer’) we found the new layer resulted in higher overall aboveground carbon and greater carbon variability following model initialization (Fig. 3a, b), with statistically significant differences occurring at the grid cell-scale (Fig. 3c). While these differences persisted throughout the 50-year simulation period, the difference in aboveground carbon between the new and old layer decreased by the end of the simulation period (Fig. 3c).

**Figure 4:**
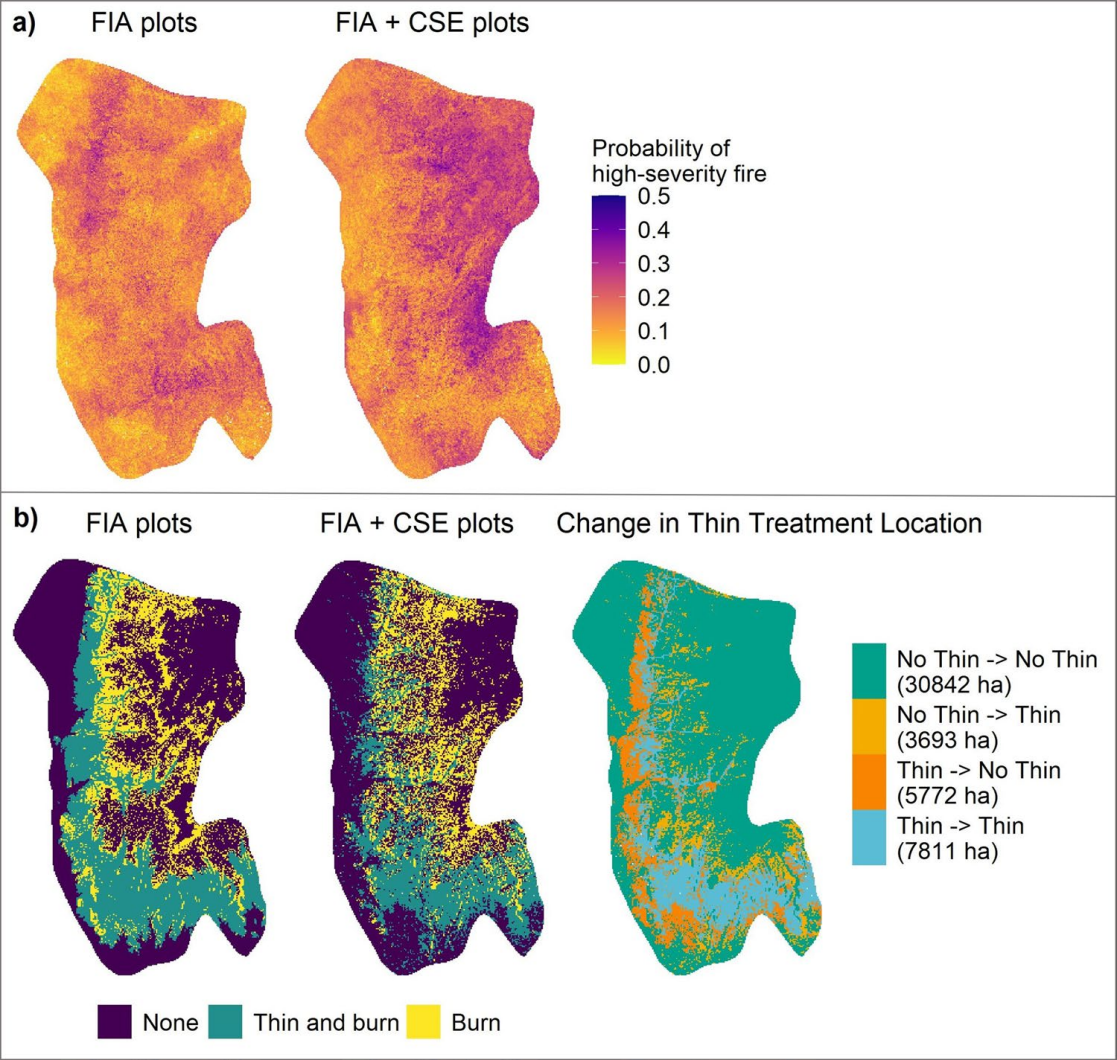
Maps of the probability of high-severity fires and the resulting treatment maps. (**a**) Probability of high severity fire in Krofcheck et al.^29^ (FIA plots) and this study (FIA + CSE plots). (**b**) The optimized treatment map from Krofcheck et al.^29^ (left), treatment map for this study (middle) and the changes in thinning treatment locations (right). Each zone indicates the type of treatment in Krofcheck et al.^29^, and what it has changed to, for example, yellow indicates areas that were not thinning in the previous study and were designated to undergo thinning in the new study.

The differences in aboveground carbon density between the new and old layers led to differences in the spatial distribution of the probability of high-severity fire (Fig. 4a). Given these differences, the distribution of thinning and prescribed fire treatments varied between the old and new layers (Fig. 4b). The carbon density and subsequent probability of high-severity fire from the new layer resulted in areas identified for thinning and burning combined, or burning alone, shifting east and up in elevation. The new treatment map also had approximately 2000 ha fewer identified as requiring thinning when compared to the old treatment map (Fig. 4b right).

**Figure 5:**
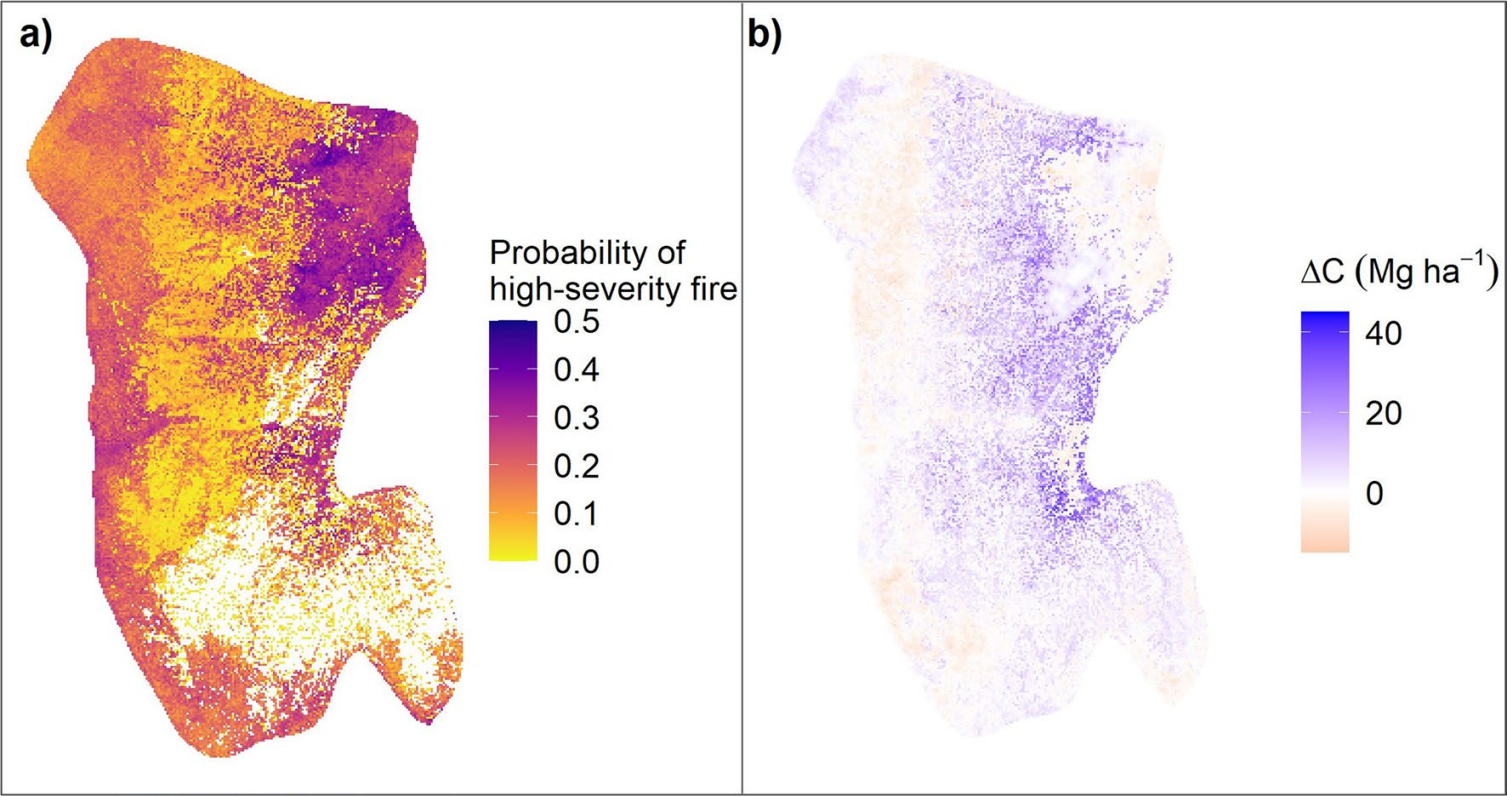
Comparison between the no management and management scenarios in this study using the new initial communities layer. (**a**) Probability of high severity fire for the management scenario. (**b**) Difference in aboveground carbon at the end of 50 years of simulation between the management and no management scenarios. Positive values indicate higher carbon in the management scenario.

We compared the simulation outputs of the management and no-management scenarios using the new initial communities layer and found that the management scenario, as expected, decreased the probability of high-severity fire where treatments were implemented (Fig. 5a). The reduction in high-severity in the management scenario led to an increase in landscape carbon storage over the 50-year simulation compared to the no-management scenario (Fig. 5b). The carbon increases were primarily in areas that were treated because the treatments reduced fire severity, while in areas that were untreated there was little difference between the management and no-management scenarios.

**Figure 6:**
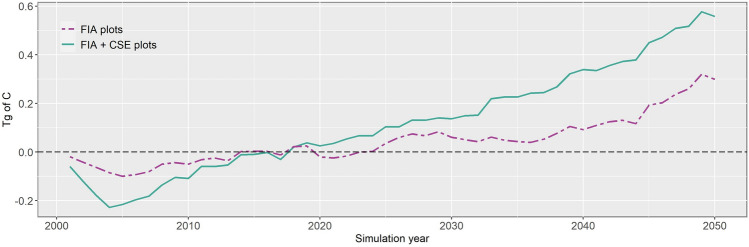
Cumulative net ecosystem carbon balance (NECB) of the optimized scenario of the previous study (Krofcheck et al.^29^) and the management scenario of this study relative to the no management scenarios of the respective studies. NECB is the balance between carbon intake from photosynthesis and carbon loss due to thinning, prescribed burns, and wildfires. Positive values indicate more carbon intake to the system in the management scenario relative to the no management scenario. Values are in Teragrams of Carbon.

Given the differences between the new and old layers in terms of probability of high-severity fire and subsequent treatment location, we compared the effects of the management scenario on cumulative Net Ecosystem Carbon Balance (NECB) relative to the no management scenario. We found similar trends for both the new and old initial communities layers. The greater carbon density in simulations using the new layer resulted in larger decreases from thinning and burning treatments early in the simulation period compared to simulations using the old layer (Fig. 6). Transition to positive cumulative NECB relative to no-management scenario occurred faster in simulations using the new layer (~ 18 years) than in simulations using the old layer (~ 24 years). This difference is due to the fact that the landscape carbon density is higher for simulations with the new layer.

## Discussion

The increased risk of high-severity wildfire due to a history of fire-exclusion and ongoing climate change presents challenges to ecosystems and communities and the scale of the problem requires a risk-based approach to allocating forest management resources^52^. Effectively deploying management resources requires both an accurate representation of forest conditions across space and accounting for how management activities, disturbance, and climate will interact over time to alter the spatial distribution of risk. Our results suggest that increasing the number of inventory plots used to develop the initial communities layer is important for capturing variability in forest conditions and can substantially alter the optimal allocation of management activities to achieve the objective, in this case reducing the risk of high-severity wildfire.

We would expect that after enough time the distribution of simulated biomass would be similar no matter the starting conditions of the forest as climate determines the ability of each grid cell to support a certain amount of vegetation. When we compared aboveground carbon in this study and the previous, we found that while the number of grid cells with significantly different aboveground carbon between the two studies decreased over time (Fig. 3c), it did not converge within the 50-year simulation period (Fig. 3b). This emphasizes the importance of sufficient sampling to characterize landscape heterogeneity.

There is a rich literature on the influence of inventory data sample size on interpolation accuracy, which indicates that more inventory data provides increasing accuracy^53,54^. Based on the data set we had available, we found increased variability for the initial communities layers that used half or more of the 1072 CSE plots. When we initiated the model with the initial communities constructed from half or all of the CSE plots, the result was a wider range of biomass densities in terms of aboveground carbon than for any of the initial communities developed using fewer plots (Fig. 2b). This result suggests that a larger inventory plot sample size better captures variability in the system, but determining how well that variability is captured will require a validation process using inventory data not included in the interpolation.

We found that the aboveground carbon distribution for the mixed-conifer and ponderosa pine type forests remained relatively consistent no matter the number of plots used to interpolate the initial forest conditions, but the aboveground carbon of the piñon-juniper and aspen forests differed and was dependent on the number of plots. This might indicate that the diversity of mixed-conifer and ponderosa pine stands throughout the study area are well characterized by the CSE plots or are simply somewhat more homogenous, whereas piñon-juniper and aspen stands were more heterogeneous (Fig. 2a). Determining if the mixed-conifer and ponderosa pine forests are in fact well-represented by this particular population of inventory plots will require sampling throughout the range of these forest types within the study area. The variability in aboveground carbon of aspen and piñon-juniper on the landscape between different initial communities is due to our approach to selecting the population of inventory plots for each interpolation. When reducing the number of plots from which we interpolated the initial forest conditions, we selected the number of plots for each forest type proportional to the prominence of that forest type on the landscape. Since aspen comprises a small fraction of the total forest cover within the landscape, we used few aspen plots for the interpolations that used fewer than half of the inventory plots. This is the most parsimonious explanation for the spread and variability in aboveground carbon for the aspen plots.

Simulation models have long been used in forest management planning. However, much of the work has focused on the stand-scale because it is the typical management unit. Managing landscapes for disturbance processes like fire requires that we plan at the scale of the disturbance. Forest landscape simulation models such as LSim and LANDIS-II can simulate disturbance processes at the appropriate scale and are useful for understanding how decision-making influences outcomes at the landscape scale^55,56^ but actionable model output at the sub-landscape scale requires an improved representation of initial conditions. The long-term goal of studies like this one and others is to develop a useful tool that would allow locating potential high-risk areas that would then be targeted for treatment by forest managers. To achieve this, it is necessary to reduce potential sources of error such that outputs of the simulations would become operational guides for decision making.

While our initial communities layer using more inventory data captured a wider range of biomass density and variability, we were only able to capitalize on a subset of the inventory plots. Although we had an additional 1072 CSE plots, they represented 111 stands and the coordinates associated with each plot were the centroid of the stand. The lack of plot-specific geographic coordinates meant that we were only able to use 111 plots (one for each stand) in the interpolation. When we examined using randomly selected plots from within each stand, we found little change in aboveground carbon. This was expected as stands are delineated to represent contiguous forest communities of similar structure. Additionally, the new CSE plots were all concentrated in the northern part of the fireshed and mostly sampled ponderosa pine and mixed-conifer forest as these data were collected to support environmental analysis and planning for a project area. While this kind of sampling can be beneficial, previous studies have pointed to the importance of sampling the entire range of species occurrences to better capture the variability when interpolating across the area of interest^57–59^. Our results demonstrate the need to ensure different vegetation types are adequately sampled. We found that biomass in the northeastern part of the study area varied with the number of plots used to construct the initial communities layer. This part of the study area is a piñon-juniper woodland and was under-represented in the inventory plots, therefore, interpolating a smaller sample of plots increased the chance that this vegetation type was poorly represented. Further, aboveground biomass in the southern part of the study area had little change regardless of the number of inventory plots used for the initial communities layer (Fig. 2c). We suspect this limited change in biomass is a result of the CSE plots being concentrated in the northern part of the study area and, in reality, there is probably greater variability in the southern part of the study area. Fully quantifying the uncertainty in the initial communities layer will require collecting inventory data across the study area and quantifying the contribution to uncertainty of both the number and location of inventory plots.

The practical importance of an accurate initial communities layer was also reflected in the different treatment maps that resulted from our initial communities layer and the one from Krofcheck et al.^29^. Our use of additional plot data to interpolate our initial communities layer captured a wider range of biomass conditions. The increased variability in biomass was important because it changed carbon and water fluxes, light availability, and growing conditions. These changes in vegetation altered the available fuels, which affected fire location severity. This resulted in large changes in the geographic location of areas with a high probability of high-severity fire relative to the analysis of Krofcheck et al.^29^. Furthermore, the total area that met the thinning criteria was lower in this study, which is important in terms of costs and resource allocation necessary to mitigate high-severity fire risk.

Our use of more inventory plots with a wider range of carbon density than the FIA plots alone to develop the initial communities layer also influenced carbon dynamics in the study area throughout the 50-year simulation (Fig. 6). The higher overall biomass across the landscape in this study compared to Krofcheck et al.^29^ was apparent in the first 5 years of the simulations, when more biomass was being removed by thinning from each grid cell, leading to a greater initial loss of carbon from the system. However, our landscape transitioned to a positive net ecosystem carbon balance (NECB) faster than in the previous study because the inclusion of additional inventory data and the subsequent interpolation resulted in fewer grid cells requiring thinning. Prior studies have demonstrated the importance of treatment size and intensity and the probability of wildfire on NECB^18,48,60^. Our results demonstrate the sensitivity of the outcome to the underlying data used to develop the initial communities layer.

The differences between our results evaluating the influences of different numbers of inventory plots on variability in the initial communities layer and differences between our results and those of Krofcheck et al.^29^ demonstrate the need for inventory data that capture the range of variability within the landscape. However, before our simulation output can be used in the management planning process to identify locations requiring treatment, we will need additional inventory data from areas throughout the extent of the study area to quantify the uncertainty in the initial communities layer as a function of the distribution of inventory data and the sampling intensity. Having the ability to estimate the uncertainty associated with the inventory data would help determine the sampling intensity necessary for using model output to support treatment planning. If the CSE data had plot-specific geographic coordinates, we could have estimate uncertainty due to sampling intensity for the northern portion of the landscape. At a minimum, future inventory sampling should include the geographic coordinates for every plot to help support future analyses.

## Conclusions

The frequency and severity of wildfires is predicted to increase as the climate gets hotter and drier, but mitigating these events is possible by restoring ecologically appropriate fire. Given the size of the area in the Southwestern US that has missed multiple fire return intervals, there is more area requiring management than there are resources to support management. Forest landscape models can help identify landscape positions with the highest probability of high-severity wildfire, but their utility for management planning is based on how well the model represents actual conditions. Operationalizing forest landscape models that have largely been research tools to-date will require forest managers and researchers working collaboratively to both inform forest inventory sample design and to determine the amount of uncertainty in model output that is acceptable when using model outputs to inform decision-making.

### Supplementary Information

Supplementary Figures.

## Data Availability

The data is available for review at: 10.5061/dryad.t4b8gtj62

## References

[1] Allen CD (2002). Ecological restoration of southwestern ponderosa pine ecosystems: A broad perspective. Ecol. Appl..

[2] Fule PZ, Covington WW, Moore MM (1997). Determining reference conditions for ecosystem management of southwestern Ponderosa pine forests. Ecol. Appl..

[3] Knapp EE, Skinner CN, North MP, Estes BL (2013). Long-term overstory and understory change following logging and fire exclusion in a Sierra Nevada mixed-conifer forest. For. Ecol. Manag..

[4] Moore MM, Huffman DW, Fule PZ, Covington WW, Crouse JE (2004). Comparison of historical and contemporary forest structure and composition on permanent plots in southwestern Ponderosa pine forests. For. Sci..

[5] Taylor AH (2014). Changes in forest structure, fuels and potential fire behaviour since 1873 in the Lake Tahoe Basin, USA. Appl. Veg. Sci..

[6] Allen CD (2010). A global overview of drought and heat-induced tree mortality reveals emerging climate change risks for forests. For. Ecol. Manag..

[7] Williams PA (2013). Temperature as a potent driver of regional forest drought stress and tree mortality. Nat. Clim. Change.

[8] Goodwin MJ, Zald HSJ, North MP, Hurteau MD (2021). Climate-driven tree mortality and fuel aridity increase wildfire’s potential heat flux. Geophys. Res. Lett..

[9] Stephens SL (2018). Drought, tree mortality, and wildfire in forests adapted to frequent fire. BioScience.

[10] Singleton MP, Thode AE, Sánchez-Meador AJ, Iniguez JM (2019). Increasing trends in high-severity fire in the southwestern USA from 1984 to 2015. For. Ecol. Manag..

[11] Westerling AL (2016). Increasing western US forest wildfire activity: Sensitivity to changes in the timing of spring. Philos. Trans. R. Soc. B Biol. Sci..

[12] Stephens SL, Ruth LW (2005). Federal forest-fire policy in the United States. Ecol. Appl..

[13] Krofcheck DJ, Hurteau MD, Scheller RM, Loudermilk EL (2018). Prioritizing forest fuels treatments based on the probability of high-severity fire restores adaptive capacity in Sierran forests. Glob. Change Biol..

[14] North M, Collins BM, Stephens S (2012). Using fire to increase the scale, benefits, and future maintenance of fuels treatments. J. For..

[15] Agee JK, Skinner CN (2005). Basic principles of forest fuel reduction treatments. For. Ecol. Manag..

[16] North MP (2021). Pyrosilviculture needed for landscape resilience of dry western United States Forests. J. For..

[17] Safford HD, Stevens JT, Merriam K, Meyer MD, Latimer AM (2012). Fuel treatment effectiveness in California yellow pine and mixed conifer forests. For. Ecol. Manag..

[18] Krofcheck DJ, Hurteau MD, Scheller RM, Loudermilk EL (2017). Restoring surface fire stabilizes forest carbon under extreme fire weather in the Sierra Nevada. Ecosphere.

[19] McIver JD (2012). Ecological effects of alternative fuel-reduction treatments: Highlights of the National Fire and Fire Surrogate study (FFS). Int. J. Wildland Fire.

[20] Shive KL, Sieg CH, Fulé PZ (2013). Pre-wildfire management treatments interact with fire severity to have lasting effects on post-wildfire vegetation response. For. Ecol. Manag..

[21] York RA, Noble H, Quinn-Davidson LN, Battles JJ (2021). Pyrosilviculture: Combining prescribed fire with gap-based silviculture in mixed-conifer forests of the Sierra Nevada. Can. J. For. Res..

[22] Collins BM (2009). Interactions among wildland fires in a long-established sierra nevada natural fire area. Ecosystems.

[23] Hurteau MD, Hungate BA, Koch GW, North MP, Smith GR (2013). Aligning ecology and markets in the forest carbon cycle. Front. Ecol. Environ..

[24] Jones GM (2022). Forest restoration limits megafires and supports species conservation under climate change. Front. Ecol. Environ..

[25] Latif QS, Cannon JB, Chabot EJ, Sparks RA (2022). Simulated treatment effects on bird communities inform landscape-scale dry conifer forest management. Ecol. Appl..

[26] Smith HG, Sheridan GJ, Lane PNJ, Nyman P, Haydon S (2011). Wildfire effects on water quality in forest catchments: A review with implications for water supply. J. Hydrol..

[27] Ager AA, Vaillant NM, Finney MA (2010). A comparison of landscape fuel treatment strategies to mitigate wildland fire risk in the urban interface and preserve old forest structure. For. Ecol. Manag..

[28] Finney MA (2007). Simulation of long-term landscape-level fuel treatment effects on large wildfires. Int. J. Wildland Fire.

[29] Krofcheck DJ, Remy CC, Keyser AR, Hurteau MD (2019). Optimizing forest management stabilizes carbon under projected climate and wildfires. J. Geophys. Res. Biogeosci..

[30] Ager AA, Barros AMG, Day MA (2022). Contrasting effects of future wildfire and forest management scenarios on a fire excluded western US landscape. Landsc. Ecol..

[31] Ager, A. A., Vaillant, N. M. & McMahan, A. Restoration of fire in managed forests: A model to prioritize landscapes and analyze tradeoffs. *Ecosphere* **4**, art29 (2013).

[32] Wei Y, Rideout D, Kirsch A (2008). An optimization model for locating fuel treatments across a landscape to reduce expected fire losses. Can. J. For. Res..

[33] Petter G (2020). How robust are future projections of forest landscape dynamics? Insights from a systematic comparison of four forest landscape models. Environ. Model. Softw..

[34] Stephens SL (2022). Mass fire behavior created by extensive tree mortality and high tree density not predicted by operational fire behavior models in the southern Sierra Nevada. For. Ecol. Manag..

[35] Remy CC (2019). Integrating species-specific information in models improves regional projections under climate change. Geophys. Res. Lett..

[36] Thornton, P. E. *et al.* Daymet: Daily surface weather data on a 1-km grid for north america, version 2. 10.3334/ORNLDAAC/1219 (2014).

[37] Roberts DW, Cooper SV (1989). Concepts and techniques of vegetation mapping. Land Classif Based Veg. Appl. Resour. Manag..

[38] Evans, J. S. & Murphy, M. A. rfUtilities. R package version 2.1-3, https://cran.r-project.org/package=rfUtilities (2018).

[39] Crookston NL, Finley AO (2008). yaImpute: An R package for kNN imputation. J. Stat. Softw..

[40] R Core Team. *R: A Language and Environment for Statistical Computing* (R Foundation for Statistical Computing, 2021).

[41] Scheller RM (2007). Design, development, and application of LANDIS-II, a spatial landscape simulation model with flexible temporal and spatial resolution. Ecol. Model..

[42] de Bruijn A (2014). Toward more robust projections of forest landscape dynamics under novel environmental conditions: Embedding PnET within LANDIS-II. Ecol. Model..

[43] Aber J (1995). Predicting the effects of climate change on water yield and forest production in the northeastern United States. Clim. Res..

[44] Gustafson EJ (2015). Integrating ecophysiology and forest landscape models to improve projections of drought effects under climate change. Glob. Change Biol..

[45] Kattge J (2011). TRY—a global database of plant traits. Glob. Change Biol..

[46] Sturtevant BR, Scheller RM, Miranda BR, Shinneman D, Syphard A (2009). Simulating dynamic and mixed-severity fire regimes: A process-based fire extension for LANDIS-II. Ecol. Model..

[47] Forestry Canada Fire Danger Group. Development and structure of the Canadian Forest Fire Behaviour Prediction System. In *For. Can. Fire Danger Group Inf. Rep. ST-X-3* (1992).

[48] Hurteau MD (2016). Restoring forest structure and process stabilizes forest carbon in wildfire-prone southwestern ponderosa pine forests. Ecol. Appl..

[49] Syphard AD (2011). Simulating landscape-scale effects of fuels treatments in the Sierra Nevada, California, USA. Int. J. Wildland Fire.

[50] Gustafson EJ, Shifley SR, Mladenoff DJ, Nimerfro KK, He HS (2000). Spatial simulation of forest succession and timber harvesting using LANDIS. Can. J. Forest Res..

[51] Hurteau MD, Stoddard MT, Fulé PZ (2011). The carbon costs of mitigating high-severity wildfire in southwestern ponderosa pine. Glob. Change Biol..

[52] Miller C, Ager AA (2013). A review of recent advances in risk analysis for wildfire management. Int. J. Wildland Fire.

[53] Fassnacht FE (2014). Importance of sample size, data type and prediction method for remote sensing-based estimations of aboveground forest biomass. Remote Sens. Environ..

[54] Nowak D, Walton J, Stevens J, Crane D, Hoehn R (2008). Effect of plot and sample size on timing and precision of urban forest assessments. Arboric. Urban For..

[55] Young JD, Ager AA, Thode AE (2022). Using wildfire as a management strategy to restore resiliency to ponderosa pine forests in the southwestern United States. Ecosphere.

[56] Liang S, Hurteau MD, Westerling AL (2018). Large-scale restoration increases carbon stability under projected climate and wildfire regimes. Front. Ecol. Environ..

[57] Asner GP (2012). A universal airborne LiDAR approach for tropical forest carbon mapping. Oecologia.

[58] Zald HSJ (2014). Influence of lidar, Landsat imagery, disturbance history, plot location accuracy, and plot size on accuracy of imputation maps of forest composition and structure. Remote Sens. Environ..

[59] Zolkos SG, Goetz SJ, Dubayah R (2013). A meta-analysis of terrestrial aboveground biomass estimation using lidar remote sensing. Remote Sens. Environ..

[60] Loudermilk EL, Scheller RM, Weisberg PJ, Kretchun A (2017). Bending the carbon curve: Fire management for carbon resilience under climate change. Landsc. Ecol..

