# Supplementary material for: Estimating the influence of field inventory sampling intensity on forest landscape model performance for determining high-severity wildfire risk
Source: Sci Rep. 2024 Feb 6;14:3073. doi: 10.1038/s41598-024-53359-8 (PMC10847129; doi:10.1038/s41598-024-53359-8)
Supplement: Supplementary file 1 — Supplementary Figures. [file 41598_2024_53359_MOESM1_ESM.pdf]

Title: Estimating the influence of field inventory sampling intensity on forest landscape model performance for determining high-severity wildfire risk

Authors: *Hagar Hecht[1,4], Dan J. Krofcheck[2,4], Dennis Carril[3], Matthew D. Hurteau[4]

[1] Spatial Informatics Group Natural Assets Lab, Pleasanton, California, USA

[2] Sandia National Laboratory, Albuquerque, New Mexico, USA

[3] US Forest Service, Santa Fe National Forest, Santa Fe, New Mexico, USA

[4] Department of Biology, University of New Mexico, Albuquerque, New Mexico, USA

*Corresponding Author: Hagar Hecht, hechtit@gmail.com

Supplementary Figures

Figure S1

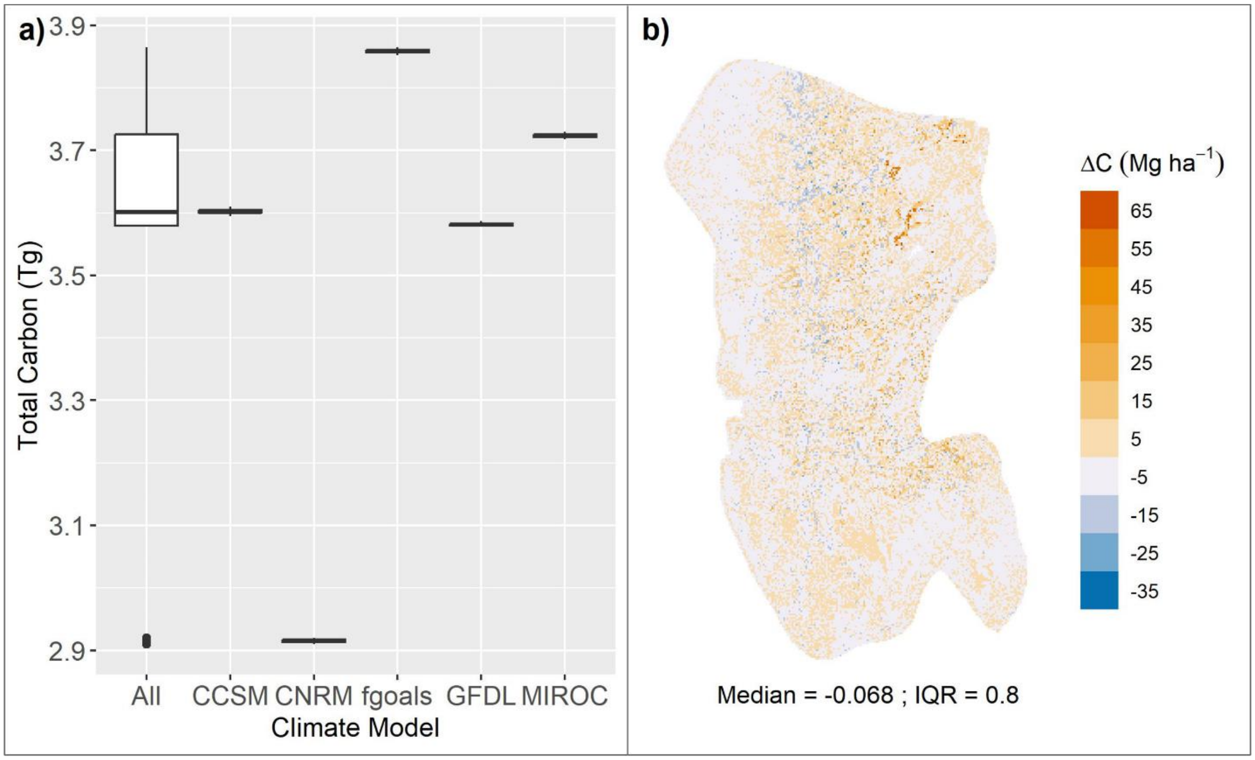

Biomass distribution for initial communities layers derived from random CSE plots selected from the same USFS measured stands. a) Boxplot of total aboveground carbon at year 1 in teragrams summed across the landscape for all climate models and for each of the climate models separately. Climate models are Community Climate System Model (CCSM), Centre National de Recherches Météorologiques (CNRM), Flexible Global Ocean-Atmosphere-Land System Model (FGOALS), Geophysical Fluid Dynamics Laboratory (GFDL), and Model for Interdisciplinary Research on Climate (MIROC5-ESM 2). b) Difference in aboveground carbon at year 1 between the primary layer and the averaged aboveground carbon of all 155 simulations derived from all 31 initial communities layers.

Figure S2

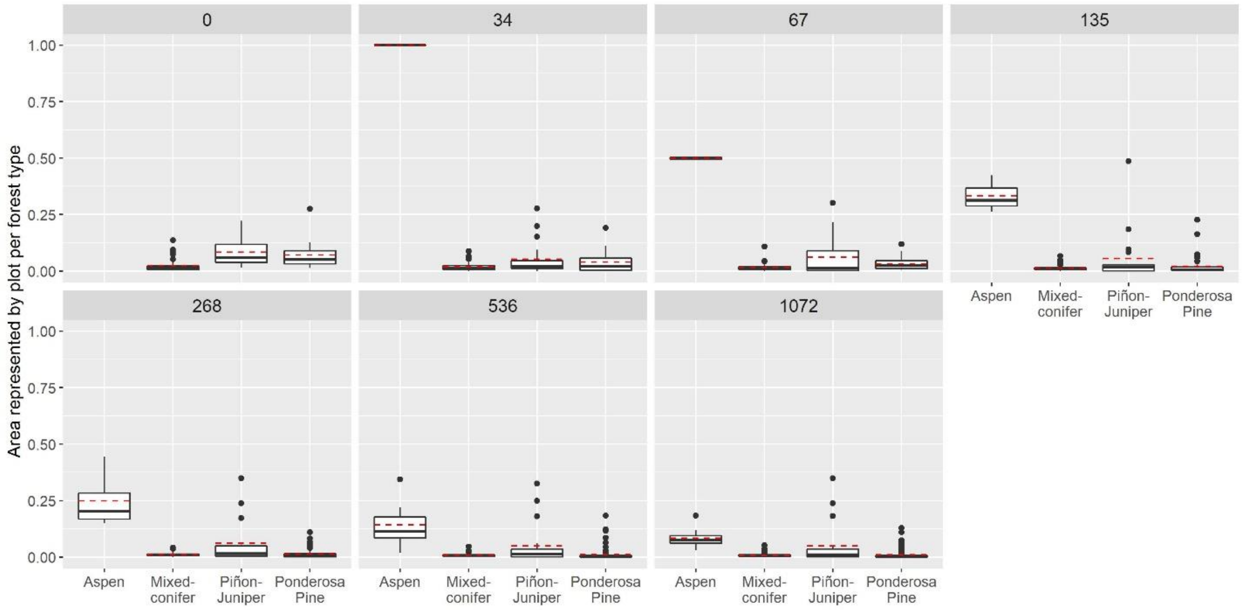

Plot sampling intensity. Area that each sampled plot represents of the corresponding forest type. Title is the number of plot sample size, red lines are the mean.
